# Effect of Restricted Preen-Gland Access on Maternal Self Maintenance and Reproductive Investment in Mallards

**DOI:** 10.1371/journal.pone.0013555

**Published:** 2010-10-27

**Authors:** Mathieu Giraudeau, Gábor Á. Czirják, Camille Duval, Vincent Bretagnolle, Cyril Eraud, Kevin J. McGraw, Philipp Heeb

**Affiliations:** 1 Laboratoire Évolution et Diversité Biologique, UMR 5174, Centre National de la Recherche Scientifique - Université Paul Sabatier, Toulouse, France; 2 Department of Infectious Diseases, Faculty of Veterinary Medicine, University of Agricultural Sciences and Veterinary Medicine, Cluj-Napoca, Romania; 3 Centre d'Etudes Biologiques de Chizé, UPR 1934, Centre National de la Recherche Scientifique, Beauvoir-sur-Niort, France; 4 Office National de la Chasse et de la Faune Sauvage, Direction des Etudes et de la Recherche, Centre National d'Etude et de Recherche Appliquée Avifaune Migratrice, Villiers-en-Bois, France; 5 School of Life Sciences, Arizona State University, Tempe, Arizona, United States of America; Institut Pluridisciplinaire Hubert Curien, France

## Abstract

**Background:**

As egg production and offspring care are costly, females should invest resources adaptively into their eggs to optimize current offspring quality and their own lifetime reproductive success. Parasite infections can influence maternal investment decisions due to their multiple negative physiological effects. The act of preening – applying oils with anti-microbial properties to feathers – is thought to be a means by which birds combat pathogens and parasites, but little is known of how preening during the reproductive period (and its expected disease-protecting effects) influences maternal investment decisions at the level of the egg.

**Methodology/Principal Findings:**

Here, we experimentally prevented female mallards (*Anas platyrhynchos*) from accessing their preen gland during breeding and monitored female immunoresponsiveness (e.g., plasma lysozyme concentration) as well as some egg traits linked to offspring quality (e.g., egg mass, yolk carotenoid content, and albumen lysozyme levels). Females with no access to their preen gland showed an increase in plasma lysozyme level compared to control, normally preening females. In addition, preen-gland-restricted females laid significantly lighter eggs and deposited higher carotenoid concentrations in the yolk compared to control females. Albumen lysozyme activity did not differ significantly between eggs laid by females with or without preen gland access.

**Conclusion/Significance:**

Our results establish a new link between an important avian self-maintenance behaviour and aspects of maternal health and reproduction. We suggest that higher yolk carotenoid levels in eggs laid by preen-gland-restricted females may serve to boost health of offspring that would hatch in a comparatively microbe-rich environment.

## Introduction

Parasites (e.g. bacteria, ectoparasites, viruses) can markedly reduce host fitness by continually draining resources [1]. Thus, hosts are favoured by selection to develop physiological, behavioural or immunological responses to combat parasite pressures [2]. Parasites can also be transmitted to host offspring, and selection should favour maternal responses that extend protection against parasites to eggs, embryos or chicks [1]. One line of maternal defence involves the deposition of non-genetic resources into eggs (e.g. antibodies, nutrients), where they influence strongly offspring health, growth, survival and phenotype [3]. However, as the deposition of these egg resources can be costly, females are faced with trading-off offspring quality against their own survival and reproductive prospects throughout life [4].

In birds, females deposit immune factors in the yolk and albumen to protect embryos with poorly developed immune systems from harmful pathogens and to enhance their development [5], [6], [7], [8], [9]. For example, lysozyme in egg albumen is a major component of maternal innate immunity in birds [10], [11]. This family of enzymes acts by digesting peptidoglycans of bacterial cell walls and thus protecting the embryo from harmful bacteria that penetrate the eggshell [11], [12]. In addition, females allocate varying amounts of carotenoids into yolk, which can quench free radicals produced during early growth and allow proper functioning of the embryonic immune system [13], [14], [8]. Carotenoids also can boost maternal health status [15]. As these compounds cannot be manufactured *de novo* by birds and must be extracted from the diet [16], mothers face allocation trade-offs between self-maintenance and deposition in eggs.

Another critical trait by which females influence offspring survival is egg size. Several studies demonstrate that egg size is related to hatching success [17], [18], early nestling survival [19], nestling condition [20] or fledgling survival [21]. For example, in mallards (*Anas platyrhynchos*), young hatched from larger eggs are better able to survive during the few days immediately after hatching [19], [22], [23].

Among the pathogens to which eggs or newly hatched animals may be exposed, there is the community of bacteria on the feathers of parents [24], [25], [26] or in the nests [27], [28] that can be transmitted to chicks or eggs by contact. These bacteria can influence bird metabolism and immune status, since during maintenance behaviour, birds ingest feather-associated bacteria that are then found in their digestive tracts [29]. Such maintenance behaviour includes preening (or direct application of preen oil to feathers using the bill), which is thought to constitute a first line of defence against parasites [30], [31], [32], [33]. Preen oil (also known as urogygial gland secretions) is composed of a mixture of aliphatic monoester waxes, fatty acids and monohydroxy wax-alcohols [34], and there are at least three modes by which preen oil could influence feather-degrading bacteria (FDB; see [26]). First, preen oil may simply form a physical barrier that prevents FDB from reaching the feather surface [33]. Second, chemicals in preen oil could have anti-microbial actions [32], [35]. Third, antibiotic-producing symbiotic bacteria could be cultivated within the uropygial gland and preened onto feathers [36]. In preliminary studies, we found that deprivation of preen gland access in mallards led to a significant change in the structure of plumage bacterial communities (Giraudeau *et al.* unpublished data). In addition, we found that surgical removal of the preen gland in adult house sparrows (*Passer domesticus*) led to an increase in the abundance of non-feather degrading bacteria on the plumage (Czirjak *et al.* unpublished data). These results show that bacterial communities change when birds do not have access to their preen gland.

Despite the potential importance of preen oil for limiting feather-associated bacterial growth, the effect of preening behavior on immune status and breeding decisions has been seldom considered to date. The goal of this study was thus to examine, for the first time, how preening behavior (and its expected disease-protecting effects) during the reproductive period influences female immunoresponsiveness and parental investment decisions at the level of the egg. We used an experimental approach by blocking preen gland access [37] in one group of captive female mallards, while another group acted as unmanipulated, control animals. An assumption of our study is that having restricted access to the preen gland elevated maternal exposure to and accumulation of environmental microorganisms [32] and altered female immunoresponsiveness. We measured lysozyme concentration in maternal plasma to assess if our treatment induced an innate constitutive immune response (i.e. higher lysozyme concentration [38], [39], [11]). In addition, we measured egg size, clutch size, yolk carotenoid concentration and albumen lysozyme concentration as indices of maternal investment. Because we also expected that chicks raised by mothers with no access to their preen gland would also suffer increased microbial exposure, we predicted that experimental females would lay eggs with higher carotenoid and lysozyme concentrations in order to increase embryo immunological protection [40]. In addition, we expected that experimental females would produce lighter eggs compared to control females due to the energetic costs of mounting an immune response [41]. Finally, differential allocation of lysozyme along the laying sequence was recently shown in the yellow-legged gull (*Larus michahellis*, [42]). The authors proposed that females deposit higher concentrations of lysozyme in the last egg because transmission of bacteria between eggs and from the mother may be more intense for the last egg. Thus, we predicted an increase of albumen lysozyme concentration with laying order.

## Methods

### Ethics statement

Housing conditions and experimental procedures were carried out in compliance with European legal recruitment and national permissions (ETS123). Moreover, we received approval (ID: A79001) from the French ethics institutions (Préfecture des Deux-Sèvres, Direction de l'Environnement et des Relations avec les collectivités locales).

### Experimental procedure

We conducted our experiment from March to April 2008 at the Centre d'Etudes Biologiques de Chizé (CEBC) in Western France, using 33 adult duck pairs (2–3 years old) descending from individuals caught in the wild in three different areas near the CEBC. Birds from these 3 areas were equally distributed in both experimental and control groups during the experiment to eliminate possible bias. The birds were kept in captive conditions at the CEBC for at least two years before the experiment, and were therefore accustomed to their aviary environment. Birds were fed with an *ad libitum* diet of water and a mixture of crushed corn, wheat and commercial duck food.

Before the beginning of the maternal investment experiment, we randomly assigned 18 females to the experimental group. Each bird was fitted with an anti-preening apparatus (APM) that was designed to prevent bill-uropygial gland contact and the spread of preen gland secretions on the feathers. The device consisted of a rubber tube of 1 cm in diameter and 2.5 cm in height, glued to the feathers and skin around the small feathered nipple of the uropygial gland. We reinforced this structure with a flexible plastic square (pierced in the middle at the ring level) glued to the plastic ring and set around the uropygial gland (Giraudeau *et al.* 2010). Ducks were visually observed with binoculars twice a week to check that the APM remained properly attached. Females from the control group did not have any device attached (N = 15). The devices were removed from all the birds once breeding was completed.

### Measurement of maternal investment

Females were housed individually in outdoor aviaries (4×4 meters) for about two weeks before the start of the experiment. One male was then randomly assigned to each female and pairs were housed together until females had finished laying the entire clutch. Eggs were collected on the day of laying, weighed, and each collected egg was then replaced with a dummy egg to induce females to lay a normal clutch. Then, three eggs per clutch – the first, middle and last in the sequence – were carefully opened over sterile Petri dishes and immediately frozen (at −20°C) until determination of yolk carotenoid concentrations and albumen lysozyme concentration. Females were captured one week before they were housed individually and one week after the laying of the last egg of the clutch for blood sampling and biometric measurements (tarsus length and mass). We drew 500 µl of whole blood from each bird through the alar vein with a heparinized syringe and immediately placed the sample on ice until centrifugation (3 min at 10 000 rpm). Plasma was then frozen at −80°C for later analysis of lysozyme and carotenoids.

### Assessment of egg and plasma compounds

To measure lysozyme concentration in albumen or plasma, we used the lysoplate assay method of Osserman and Lawlor (1966) [43]: 25 µL plasma or albumen were inoculated in the test holes of a 1% agar gel (A5431, Sigma) containing 50 mg/100 ml lyophilized *Micrococcus lysodeikticus* (M3770, Sigma), a bacteria which is particularly sensitive to lysozyme concentration. Crystalline hen egg white lysozyme (L6876, Sigma) was used to prepare a standard curve in each plate. Plates were incubated at room temperature (25–27°C) for 18 h. During this period, as a result of bacterial lysis, a clear zone developed in the area of the gel surrounding the sample inoculation site. The diameters of the cleared zones are proportional to the log of the lysozyme concentration. This area was measured using digital callipers, and converted on a semilogarithmic plot into hen egg lysozyme equivalents (HEL equivalents, expressed in µg/mL) according to the standard curve. We found a highly significant correlation between two independent measurements of the same sample, indicating that our method of quantifying lysozyme concentration was repeatable between assays (F_1,28_ = 64791; P<10^−8^).

Methods for plasma and yolk carotenoid extractions and HPLC analyses follow those described in McGraw and Ardia (2003) [15]. Due to technical problems, albumen lysozyme concentration was measured from only 18 of the clutches (APM = 9, Control = 7) and yolk carotenoid concentration from 27 of the clutches (APM = 15, Control = 12).

### Statistical analyses

Lysozyme data were log transformed to normalise them. All other data met assumptions of parametric statistics. We performed T-tests on female morphological traits to ensure that they did not differ between treatment groups. We examined effects of female treatment on body mass, body condition and plasma lysozyme concentration through repeated-measures analyses of variance (rmANOVAs). Body condition was expressed as the residual mass from a linear regression relating body mass to tarsus length.

We calculated mean egg mass for each clutch and performed T-tests to examine the effect of female treatment on this variable. We performed repeated-measures analyses of variance (rmANOVAs) to test if preen-gland treatment, laying order and the interaction between these two factors influenced yolk carotenoid concentration and albumen lysozyme concentration. Finally, we performed linear regression to examine if female physiological status (body condition, plasma carotenoid and lysozyme) predicted egg characteristics (mean mass, yolk carotenoid and albumen lysozyme per clutch). Data were analyzed using STATISTICA 6.0 software (Statsoft, Tulsa, USA).

## Results

### Effects of preen-gland treatment on females

**Figure 1:**
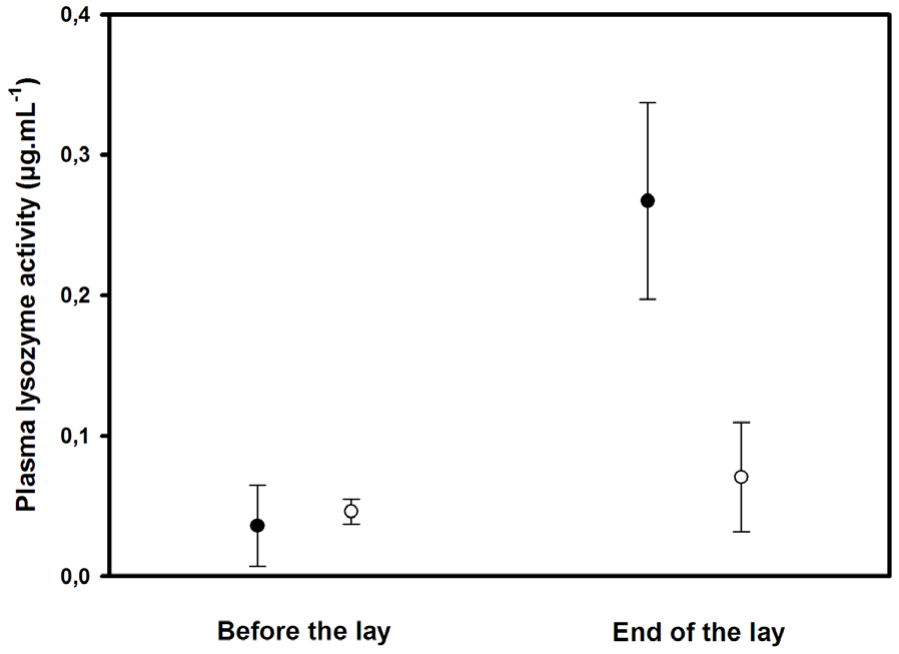
Effect of the preen-gland treatment on plasma lysozyme concentration (±SE) in breeding female mallards. White points represent females with free access to the preen gland while black points represent females for which preen gland access was blocked.

Before the start of the experiment, there were no significant differences in tarsus length (t = −0.3, P = 0.76), body mass (t = 1.4, P = 0.17), body condition (t = 1.09, P = 0.28), plasma lysozyme concentration (t = −0.24, P = 0.81) or plasma carotenoid concentration (t = 0.77, P = 0.44) between females assigned to the experimental or control groups. During the experiment, we found that females with no access to their preen gland lost more mass (F_1,31_ = 8.58, P = 0.006) and were in lower body condition at the end of the experiment (F_1,31_ = 10.36, P = 0.003) compared to control birds. Moreover, plasma lysozyme concentration increased in females without access to the preen gland, while it stayed constant in control females during the experiment (Treatment: F_1,30_ = 4.8, P = 0.04; Time*Treatment: F_1,30_ = 4.6, P = 0.04, Figure 1 and Table 1). By contrast, plasma carotenoid concentration change during the experiment was not affected by treatment (t = −0.62, P = 0.5).

**Table 1:** Egg characteristics and female immune status according to treatment (Mean and SE).

	Mean (APM/control)	SE (APM/control)
**Female mass loss during the experiment (g)**	319/237	18.53/20.78
**Female plasma lysozyme (**µ**g.mL^−1^)**		
Beggining of the experiment	0.036/0.046	0.029/0.009
End of the experiment	0.27/0.07	0.07/0.04
**Clutch size**	11.67/12.53	0.45/0.74
**Egg mass (g)**	52.66/56.11	0.83/1.21
**Yolk carotenoid concentration (µg.g^−1^)**		
First egg	57.13/47.75	6.48/8.56
Middle Egg	49.14/37.52	3.29/3.98
Last egg	50.34/40.95	4.13/3.94
**Albumen lysozyme concentration (**µ**g.mL^−1^)**		
First egg	61.4/66.57	23.47/12.51
Middle Egg	39.66/57.23	7.54/22.81
Last egg	102.82/99.96	37.23/41.48

### Maternal investment

#### Egg mass and clutch size

**Figure 2:**
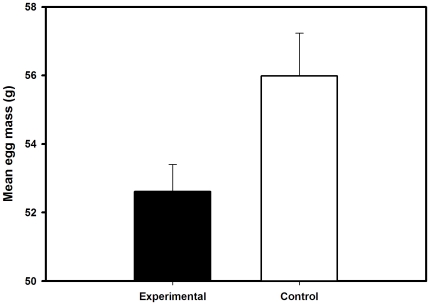
Mass of eggs (mean + SE) laid by female mallards with access or no access to their preen glands.

We found that females with no access to their preen gland laid significantly lighter eggs compared to controls (t = −2.27, P = 0.03, N = 33, Figure 2 and Table 1). In contrast, clutch size was not significantly different between the two groups of females (t = −1.04, P = 0.3, N = 33). Female body condition before laying the clutch did not influence the mass of eggs laid (F_1,31_ = 0.04, P = 0.84).

### Yolk carotenoids

**Figure 3:**
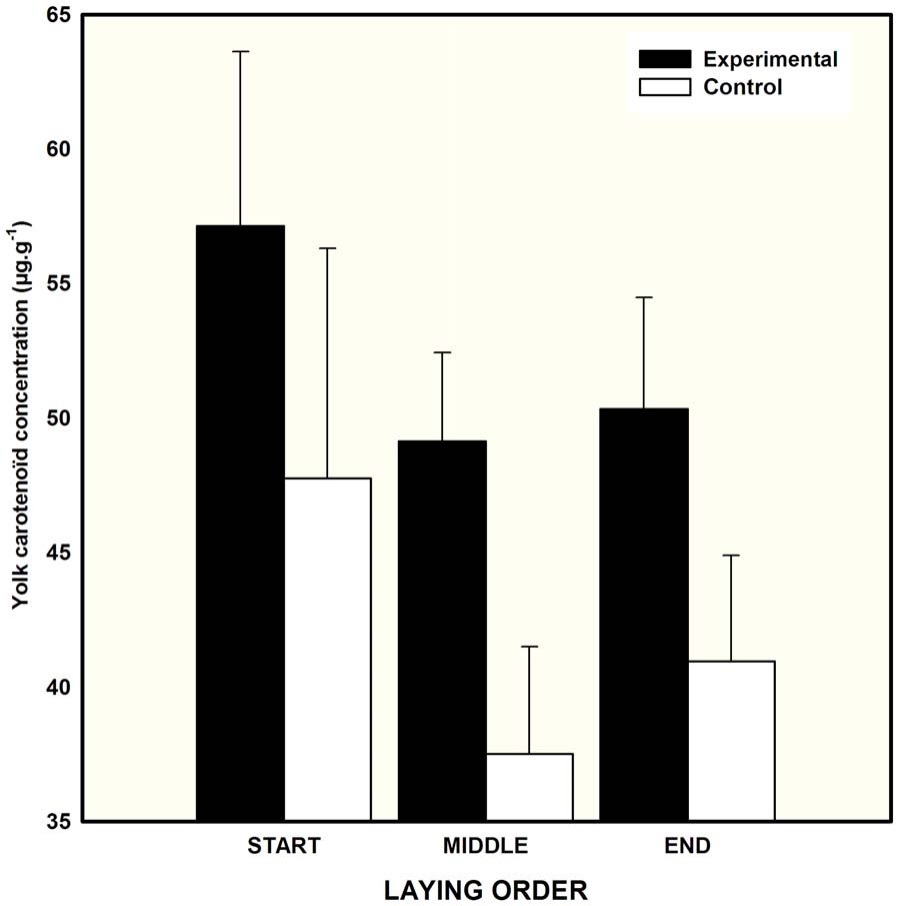
Yolk carotenoid concentration (mean + SE) of eggs laid by female mallards with access or no access to their preen glands.

We found that experimental females deposited higher concentrations of carotenoids into egg yolk than control ones (Tables 1 and 2 and Figure 3). Laying order did not influence yolk carotenoid deposition, and no significant interaction was found between egg laying order and preen-gland treatment (Table 2). Female plasma carotenoid concentrations measured before the experiment did not predict the concentration of these compounds in the yolk of eggs (mean yolk carotenoid/clutch, F_1,25_ = 0.55, P = 0.47).

**Table 2:** Effect of restricting access to the preen gland of breeding female mallards on egg characteristics.

Egg characteristics	Factors	*F*	*P*
**Albumen lysozyme concentration**	Treatment	0.05	0.81
	Laying order	3.04	0.06
	Treatment*Laying order	1.16	0.33
**Yolk carotenoid concentration**	Treatment	5.03	**0.03**
	Laying order	0.26	0.26
	Treatment*Laying order	0.21	0.81

### Albumen lysozyme

**Figure 4:**
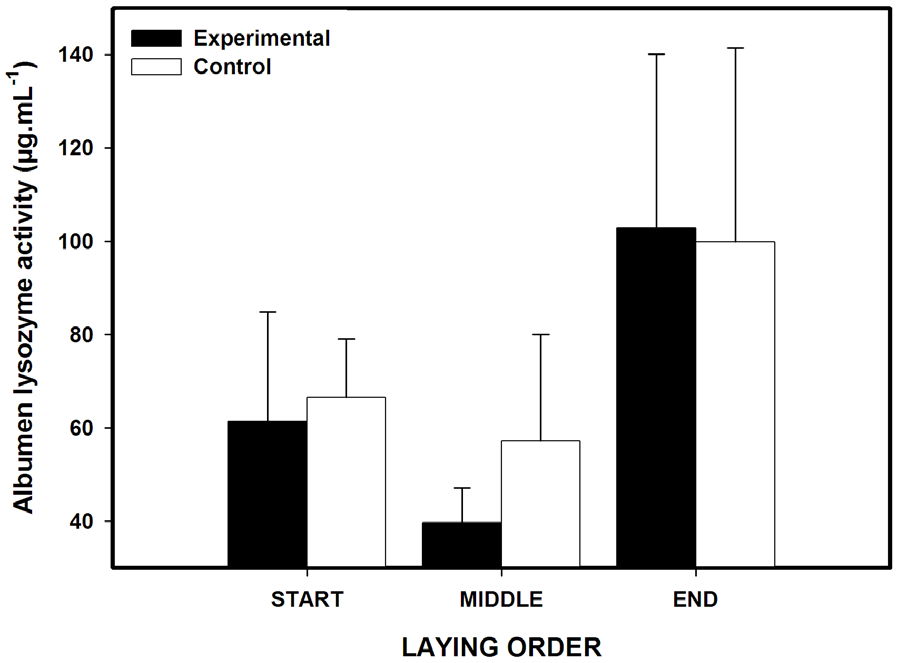
Lysozyme concentration in the albumen of eggs (mean + SE) laid by female mallards with access or no access to their preen glands.

Albumen lysozyme concentration was not influenced by maternal preen-gland treatment (Figure 4, Table 2). Also, no significant interaction existed between egg laying order and maternal treatment. However, laying order tended to influence albumen lysozyme, with lysozyme concentration increasing along the laying sequence (Tables 1 and 2 and Figure 4). Maternal plasma lysozyme concentration measured before the experiment did not predict albumen concentration of this enzyme (mean albumen lysozyme concentration/clutch, F_1,31_ = 0.26, P = 0.61). Finally, we did not find any significant covariance between mean egg mass, carotenoid concentration and lysozyme concentration (P>0.5).

## Discussion

We found that blocking preen gland access in breeding female mallards modified several aspects of their condition and health. First, our treatment increased maternal lysozyme concentration in plasma and thereby constitutive immune status. Lysozymes are a major component of innate antibacterial immunity [10], [11], acting by digesting peptidoglycans of bacterial cell walls [11]. Thus, higher plasma levels of this antimicrobial enzyme in experimental females is consistent with the fact that they were exposed to more bacteria during egg laying [38], [44], [11]. As preen oil constitutes the first line of defence against potential environmental plumage parasites and particularly feather-associated bacteria [30], [32], [33], we argue that experimental females faced higher densities of detrimental bacteria on their plumage and probably ingested them while grooming [29]. In a previous experiment on mice, inoculation of pathogenic bacteria (flagellin from *Salmonella enterica serovar Enteritidis*) led to an elevation of plasma lysozyme [44]. For the moment, these kinds of experiment seem to be lacking in birds [11] and our study provides, for the first time, information about plasma lysozyme concentration in birds subjected to a microbe-rich environment.

We also found that experimental females lost more body mass and were in worse body condition during the study than controls females. Bacterial exposure and mounting an immune response may have significant effects on energy intake and metabolism, such as an increased utilization of glucose by peripheral tissues [45]. Consistent with our results, previous studies in poultry and mammals have shown that stimulation of the immune system with LPS results in an acute reduction of body weight gain and feed intake [46], [47], [48], [41]. An alternative hypothesis could be that females with the device that did not allow them access to the preen gland were more stressed than females that had access to their preen gland. However, we have three lines of evidence that work counter to this argument. First, in a preliminary study we did not find any significant difference in time devoted to several behaviours (e.g. grooming, feeding, walking, sleeping, bathing, courtship) between birds equipped with an APM or with a control-preening mechanism (same mechanism as the APM but without the small tube that prevents bill/preen gland contact; [37]). Second, we did not find any effect of our treatment on circulating carotenoids levels, a variable that can be affected by stressful conditions [49], [50]. Third, we found increased, not decreased, deposition of yolk carotenoids by experimental females compared to control ones (see more below). Taken together, these results suggest that blocking preen gland access does not increase stress in mallards, but we cannot rule out this hypothesis. Unfortunately, we do not have blood samples available to test this idea, but in future work we suggest that measuring blood or feces corticosterone levels would be a useful addition to this line of work.

In addition to the somatic effects on mothers, we found that restricting maternal access to the preen gland altered egg investment. Experimental females deposited more carotenoids and produced lighter eggs than control females. The fact that eggs were lighter is consistent with the idea that these females were in worse condition and health and thus could devote fewer resources to eggs, as has been shown previously [51], [52], [53]. Dufva (1996) [54], for example, showed that female great tits (*Parus major*) infected with *Trypanosoma* blood parasites laid smaller eggs than non-infected females. However, the shift in carotenoid allocation differs from the results of Saino *et al.* (2002a) [13], who found that wild female barn swallows (*Hirundo rustica*) whose immune system had been experimentally challenged with an antigen deposited lower lutein concentrations in their eggs than control females. If we assume that microbe exposure was also higher in eggs and chicks of females that had no access to their preen gland, then it is possible that elevated carotenoid levels worked to boost immunity [13], [8] in comparatively pathogen-challenged offspring. It is interesting that another health-related metric – albumen lysozyme concentration – was not altered by the preen gland treatment, suggesting a quite carotenoid-specific mechanism here. Small sample size could have limited our statistical power in this analysis as well. However, it should be noted that the lysozyme patterns we obtained were very consistent and similar in relation to the laying sequence. The precise carotenoid allocation mechanism at work is far from clear; carotenoids probably were not a limiting resource in our captive study (i.e. birds were fed *ad libitum*, carotenoid levels did not decline with laying order), and treated females did not show higher circulating plasma carotenoid levels compared to controls.

In conclusion, we found evidence that breeding female mallards are somatically affected by having no access to their preen gland and also modulate their reproductive investment accordingly. Further studies should examine the proximate mechanisms and fitness consequences of maternal deposition of immune compounds in eggs as a function of pathogen exposure.
